# Recombinant Coxsackievirus A2 and Deaths of Children, Hong Kong, 2012

**DOI:** 10.3201/eid1908.121498

**Published:** 2013-08

**Authors:** Cyril C.Y. Yip, Susanna K.P. Lau, Patrick C.Y. Woo, Samson S.Y. Wong, Thomas H.F. Tsang, Janice Y.C. Lo, Wai-Kwok Lam, Chak-Chi Tsang, Kwok-Hung Chan, Kwok-Yung Yuen

**Affiliations:** The University of Hong Kong, Hong Kong, China (C.C.Y. Yip, S.K.P. Lau, P.C.Y. Woo, S.S.Y. Wong, K.-H. Chan, K.Y. Yuen);; Department of Health, Hong Kong (T.H.F. Tsang, J.Y.C. Lo, W.-K. Lam, C.-C. Tsang)

**Keywords:** coxsackievirus, coxsackievirus A2, viruses, recombination, recombinant virus, children, deaths, Hong Kong

## Abstract

A natural recombinant of coxsackievirus A2 was found in 4 children with respiratory symptoms in Hong Kong, China, during the summer of 2012. Two of these children died. Vigilant monitoring of this emerging recombinant enterovirus is needed to prevent its transmission to other regions.

Human coxsackieviruses belong to the family *Picornaviridae* and genus *Enterovirus*. These viruses are divided into groups A and B on the basis of their pathogenicity in suckling mice (flaccid paralysis caused by group A viruses and spastic paralysis caused by group B viruses) (*1*). Human infections with enteroviruses such as coxsackievirus A (CVA) are generally mild, but severe complications were more often reported for infections caused by enterovirus 71 (EV71) (*1**,**2*). We report infection of 4 children with recombinant coxsackievirus A2 in Hong Kong.

## The Study

On June 10, 2012, a previously healthy 4-year-old boy in Hong Kong had fever, cough, and rhinorrhea. His condition deteriorated rapidly and he lost consciousness. He was admitted to Pamela Youde Nethersole Eastern Hospital in Hong Kong, cardiac asystole developed, and he died <4 hours after admission. Autopsy showed areas of dull red consolidation on the upper lobes of the lungs. Postmortem histologic examination showed focal areas of alveolar damage and hyaline membrane formation with lymphocytic infiltrates. There were no gross or histologic changes indicative of myocarditis or encephalitis in any part of his brain, including the brainstem.

Tissue samples of heart, lung, spleen, and rectum, and nasopharyngeal and rectal swab specimens were positive for enterovirus by virus culture in a rhabdomyosarcoma cell line. These samples were also positive by reverse transcription PCR (RT-PCR) with pan-enterovirus primers (5′-CAAGCACTTCTGTBWCCCCGG-3′ and 5′-GAAACACGGACACCCAAAGTAGT-3′) specific for the 5′ untranslated region (5′-UTR) as described (*3**–**5*).

Because sequence analysis of the 5′-UTR amplicon showed that this enterovirus strain was closely related to other human enterovirus A strains, another set of consensus primers (5′-TGCCCACAYCARTGGATHAA-3′ and 5′-CCTGACCACTGNGTRTARTA-3′) specific for the viral protein 2–viral protein 3 region of human enterovirus A was used for typing. CVA2 strain 430895 was identified. No other pathogens were detected in these specimens.

On June 19, 2012, a previously healthy 2-month-old girl who lived in Hong Kong had an upper respiratory tract infection for 3 days. She was cyanotic and unresponsive, and showed cardiorespiratory arrest at admission to the emergency department of Queen Elizabeth Hospital in Kowloon. Despite attempted resuscitation, she died <1 hour after admission. No epidemiologic link was found between this patient and the previous patient when public health officials interviewed the parents about exposure histories. Mild perivascular lymphocytic infiltrates were observed in postmortem lung samples. No gross or histologic evidence of encephalitis or myocarditis was found at postmortem examination. Although airway samples were positive for community-acquired methicillin-resistant *Staphylococcus aureus* and cytomegalovirus, no characteristic histologic changes indicative of such infections were found in lung tissues. One intestinal sample from this patient was positive for CVA2 strain 2260165 by RT-PCR.

Two other closely related CVA2 strains were detected in 2 children with respiratory symptoms in early June 2012. A 4-year-old boy was admitted to Pamela Youde Nethersole Eastern Hospital and a 10-month-old boy was admitted to Queen Mary Hospital for fever and upper respiratory tract infection. Both patients recovered. Their CVA2 strains, 431135 and 431306, respectively, were detected by RT-PCR in nasopharyngeal aspirates.

To understand the molecular basis for the possible pathogenetic mechanism of these CVA2 strains, we analyzed their complete genome sequences. The genome sequences of the 4 CVA2 strains were amplified and sequenced as described (*4**,**5*) and deposited in GenBank under accession nos. JX867330–JX867333. The genomes of the 4 CVA2 strains are 7.4 kbp and have a G + C content of 48.8%–48.9% (excluding the 3′ polyadenylated tract). They have sequence identities of 99.6%–99.9%.

Nucleotide and amino acid sequence identities between the 4 CVA2 strains and other human enterovirus A strains were compared (Table). Sequences of the capsid region (P1) of the 4 CVA2 strains showed >81.6% nt and >96.9% aa identities with the CVA2 prototype strain Fleetwood (CVA2F), suggesting that these strains belonged to the same serotype as CVA2. The 5′-UTR and nonstructural regions (P2 and P3) of the 4 CVA2 strains had highest sequence identities with those of other human enterovirus A strains but not with CVA2F.

**Table:** Pairwise sequence identities between coxsackievirus A2 strains from 4 children Hong Kong, China, 2012, in and other representative human enterovirus A strains

Region	Nucleotide identity, %			Amino acid identity, %	
	CVA2F	Other human enterovirus A strains		CVA2F	Other human enterovirus A strains
5′-UTR	84.9–85.3	71.1–87.2		NA	NA
Polyprotein	79.3–79.4	69.1–78.4		96–96.1	76–88.4
P1	81.6–81.7	64.8–69.1		96.9–97	67.4–74.6
VP4	80.2–80.7	61.8–70.8		97.1	63.8–81.2
VP2	81.6	66.1–70.3		97.6	72.9–79.2
VP3	82.4–82.6	66.1–72.8		97.1–97.5	70.7–83.3
VP1	81.1–81.2	60.1–65.9		95.9–96.3	56.7–67.3
P2	78–78.2	70.5–83.8		96.9–97.1	78.9–98.4
2A	78–78.2	65.3–82		96	69.3–98.7
2B	73.7–74.1	66–84.2		94.9	76.8–99
2C	79.2–79.4	71.9–86.3		97.9–98.2	83–98.8
P3	77.8–77.9	72–87.8		94.4–94.6	81.1–97.9
3A	79.8–80.6	69–86.4		94.2–95.3	68.6–98.8
3B	74.2	62.1–93.9		90.9	68.2–95.5
3C	77.4	72.3–87.4		95.1	84.2–98.9
3D	77.7–77.9	72.2–89.6		94.2–94.4	82.7–98.5
3′-UTR	83.3–84.5	38.9–96.3		NA	NA

*CVA2F, coxsackievirus A prototype strain Fleetwood; UTR, untranslated region; NA, not applicable; P1, capsid protein 1; VP, viral protein; P2, nonstructural protein 2; P3, nonstructural protein 3.

**Figure 1:**
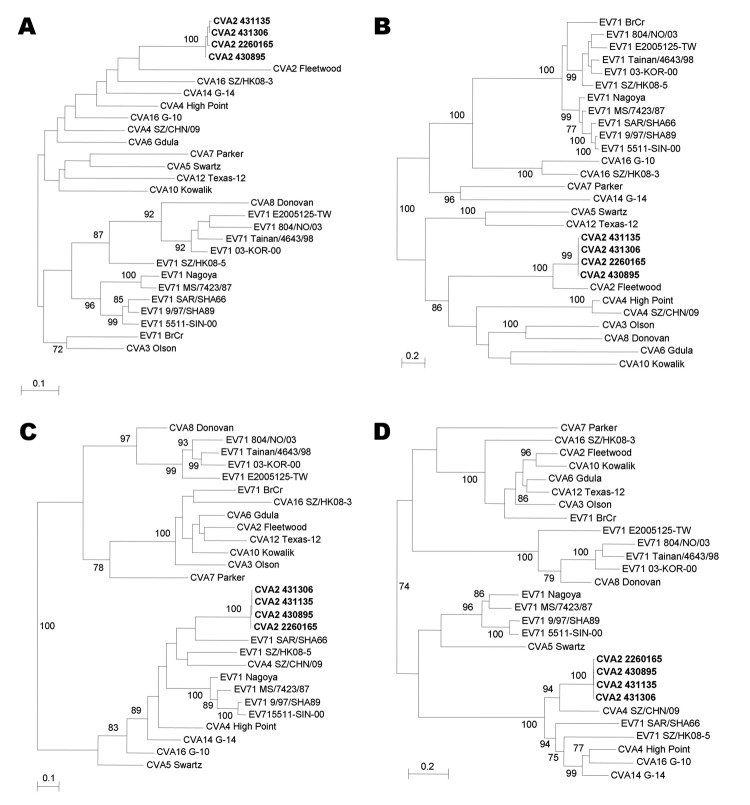
Phylogenetic trees of A) 5′-untranslated region (UTR), B) capsid protein (P1), C) nonstructural protein 2 (P2), and D) nonstructural protein 3 (P3) regions of 4 coxsackievirus A2 (CVA2) strains, Hong Kong, 2012 and other human enterovirus (EV) A strains with complete genome sequences. Trees were inferred from data by using the maximum-likelihood method with bootstrap values calculated from 1,000 trees. Sequences for 758-nt positions in each 5′-UTR, 2,595 nt positions in each P1 region, 1,734 nt positions in each P2 region, and 2,259 nt positions in each P3 region were included in the analysis. Only bootstrap values >70% are shown. Scale bars indicate estimated number of nucleotide substitutions per 5 (B and D) or 10 (A and C) nucleotides. CVA2 strains isolated in this study are indicated in **boldface**. Virus strains (GenBank accession nos.) used were CVA2 Fleetwood (AY421760), CVA3 Olson (AY421761), CVA4 High Point (AY421762), CVA4 SZ/CHN/09 (HQ728260), CVA5 Swartz (AY421763), CVA6 Gdula (AY421764), CVA7 Parker (AY421765), CVA8 Donovan (AY421766), CVA10 Kowalik (AY421767), CVA12 Texas-12 (AY421768), CVA14 G-14 (AY421769), CVA16 G-10 (U05876), CVA16 SZ/HK08–3 (GQ279368), EV71 BrCr (U22521), EV71 Nagoya (AB482183), EV71 MS/7423/87 (U22522), EV71 SAR/SHA66 (AM396586), EV71 9/97/SHA89 (AJ586873), EV71 5511-SIN-00 (DQ341364), EV71 804/NO/03 (DQ452074), EV71 Tainan/4643/98 (AF304458), EV71 03-KOR-00 (DQ341356), EV71 SZ/HK08–5 (GQ279369), and EV71 E2005125-TW (EF063152).

Phylogenetic trees were constructed by using nucleotide sequences of the 5′-UTR and P1, P2, and P3 regions of the 4 CVA2 strains and other human enterovirus A strains with complete genome sequences (Figure 1). Sequence alignment was performed by using ClustalX version 2.0 (*6*). The best evolutionary model (general time reversible + invariant sites) for each dataset was determined by using ModelGenerator (*7*). Maximum-likelihood phylogenetic trees were constructed by using PhyML version 3.0 (*8*), and bootstrap values were calculated from 1,000 trees. Phylogenetic analysis showed that the 4 CVA2 strains were most closely related to CVA2F in the 5′-UTR and P1 region. The 4 CVA2 strains clustered with EV71 subgenotype B3 strain SAR/SHA66 in the P2 region but with CVA4 strain SZ/CHN/09 in the P3 region.

**Figure 2:**
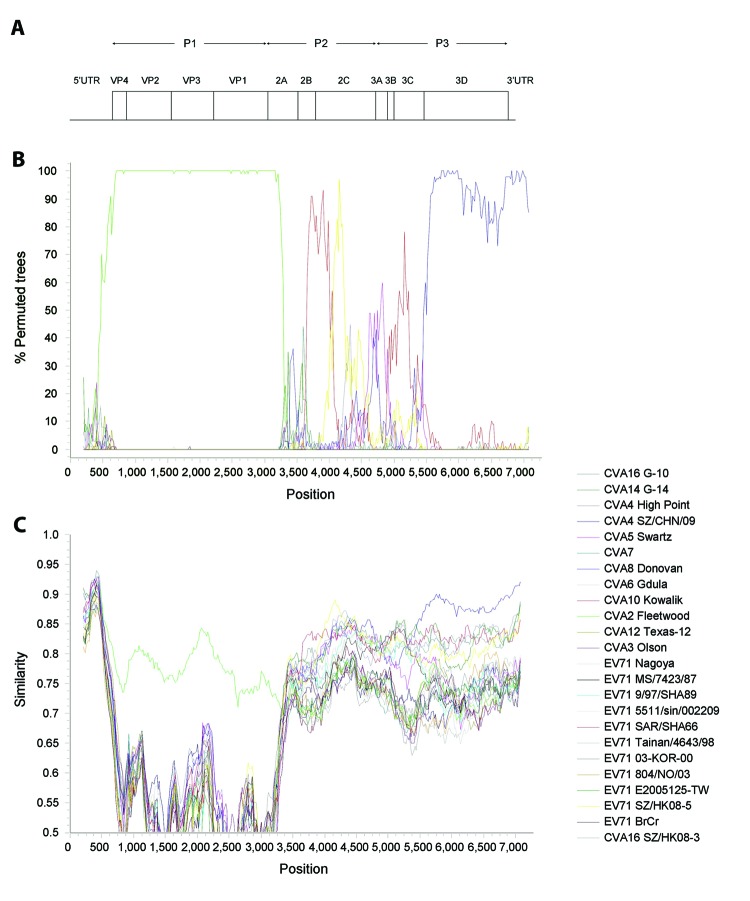
Recombination analysis of complete genome of coxsackievirus A2 (CVA2), Hong Kong, 2012. A) Genome organization. B) Bootscanning and C) similarity plot analyses were conducted by using Simplot version 3.5.1 (http://sray.med.som.jhmi.edu/SCRoftware/simplot/) (Kimura distance model; window size, 400 bp; step, 20 bp) on a gapless nucleotide alignment generated with ClustalX 2.0 (*8*) with the genome sequence of CVA2 strain 430895 as the query sequence. In panel B, green line indicates CVA2 prototype strain Fleetwood, red line indicates EV71 strain SAR/SHA66 of subgenotype B3, yellow line indicates EV71 strain SZ/HK08–5 of subgenotype C4, and blue line indicates CVA4 strain SZ/CHN/09. P1, capsid protein 1, P2, nonstructural protein 2; P3, nonstructural protein 3; UTR, untranslated region; VP, viral protein.

Further analysis was performed to identify potential recombination sites (Figure 2). Multiple sequence alignment of genomes of representative CVA2 strain 430895 and other human enterovirus A strains was generated by using ClustalX version 2.0 and edited manually. Once aligned, similarity plot and bootscan analyses were conducted by using Simplot version 3.5.1 (window size 400 bp, step 20 bp) (*9*), with the genome sequence of CVA2 strain 430895 as the query sequence.

Results showed high bootstrap supports for clustering between CVA2F and the 4 CVA2 strains at nucleotide positions 700–3400, between EV71 strain SAR/SHA66 subgenotype B3 and the 4 CVA2 strains at nt positions 3700–4030, between EV71 strain SZ/HK08–5 subgenotype C4 and the 4 CVA2 strains at nt positions 4030–4300, and between CVA4 strain SZ/CHN/09 and the 4 CVA2 strains at nt position 5700 to the 3′ end of the genome. These findings indicated that recombination events might have occurred between nt positions 3400 and 3700 (corresponding to the 2A region), at nt position 4030 in the 2B region, and between positions 5400 and 5700 (corresponding to the 3C region). Several possible recombination events were detected in other regions of the CVA2 genome but with lower bootstrap supports.

## Conclusions

Because noncapsid regions of enteroviruses are not correlated by serotype (*10*), results of phylogenetic and recombination analyses were based on the highest sequence similarity between enterovirus strains. Although recombination was evident in the 4 CVA2 strains, lack of comparative sequences indicated that the timing of recombination events was unknown, and low overall similarity to comparison sequences suggested that these events could be distant in time. As in many studies of human enterovirus A strains, lack of complete genome sequences for most serotypes, with the possible exception of EV71, limits interpretation of results for recombination analysis (*10**,**11*). In the present study, only 2 complete genome sequences of CVA4 strains (prototype strain High Point and strain SZ/CHN/09) were included in recombination analysis. Therefore, sequencing and analysis of more complete genome sequences of human enterovirus A strains, particularly CVA strains, from a wider geographic area over a longer period will provide a clearer picture of the role of recombination in this species.

We report a novel enterovirus isolated from or detected in 4 young children with severe upper respiratory tract infections in Hong Kong, 2 of whom died. This virus was characterized by complete genome sequencing as a recombinant virus of at least 3 enteroviruses (CVA2, EV71, and CVA4), and had the capsid of CVA2. Although it could not be determined whether this virus was the cause of the deaths, this report might serve to alert other investigators of circulation of a more pathogenic enterovirus.
